# CNN-LSTM-Based Damage Localization of Plate Structure

**DOI:** 10.3390/ma18092081

**Published:** 2025-05-01

**Authors:** Yajie Sun, Xiaowen Zhou, Qian Long, Mingchun Jian

**Affiliations:** School of Computer Science, Nanjing University of Information Science & Technology, Nanjing 210044, China; 202212490382@nuist.edu.cn (X.Z.); 202312490806@nuist.edu.cn (Q.L.); 202312490608@nuist.edu.cn (M.J.)

**Keywords:** convolutional neural network, long short-term memory, feature extraction, damage location

## Abstract

To address the challenges of feature extraction from time-domain signals and imprecise damage localization in conventional plate structure damage identification methods, this study proposes an innovative damage localization approach integrating Convolutional Neural Networks (CNNs) and Long Short-Term Memory (LSTM) networks. The one-dimensional signal data extracted from the aluminum plate is converted into a two-dimensional grayscale image, leveraging the advantages of CNN to accurately extract the information features of the damaged image on the aluminum plate. These extracted features are subsequently channeled into the LSTM network and the unique forgetting and memory mechanisms inherent in LSTM are employed to integrate the input feature information through a three-layer LSTM network, which is then fed into a fully connected layer for regression prediction. This method is not only an innovative application of joint deep learning methods in the field of damage detection but also accurately predicts the coordinates of the damage location, effectively overcoming the limitations of traditional damage localization methods. To validate the effectiveness of our proposed method, experiments were conducted on aluminum plates. The results demonstrate that our method shows strong performance in accurately localizing artificial damage on aluminum plates.

## 1. Introduction

Structural health monitoring (SHM) is a crucial implementation process aimed at acquiring real-time information about the design framework, performance, and health status of structures. This endeavor is paramount for improving the reliability and safety of structures and concurrently reducing maintenance costs [1,2]. With the continuous development and progress of various industries [3], plate structures, including aircraft wings, rocket shells, and stiffened composite plates [4,5], increasingly rely on composite materials. However, operational cycles, load wear, and potential collisions may result in various forms of damage to these composite materials [6,7], such as fiber fracture, interlayer separation, and material cracking [8], consequently weakening the material’s strength. Therefore, the effective monitoring of damage in various plate structures is of immense significance in driving forward the progress of science and technology. Structural health monitoring (SHM) is a critical implementation process aimed at acquiring real-time information about the design framework, performance, and health status of structures. This endeavor is paramount for enhancing the reliability and safety of structures, while simultaneously reducing maintenance costs. With the continuous development and advancement across various industries, plate structures, including aircraft wings, rocket shells, and stiffened composite plates, are increasingly reliant on composite materials. However, operational cycles, load wear, and potential collisions may result in various forms of damage to these composite materials, such as fiber fracture, interlayer separation, and material cracking, thereby compromising the material’s strength. Hence, effective monitoring of damage in various plate structures is of significant importance in driving the progress of science and technology. By employing more academic language, the expression of the text can be made more rigorous and professional, enhancing the academic and readability of the article.

Guided waves are extensively utilized in damage localization technology due to their high sensitivity to both surface and internal defects, coupled with exceptional signal transmission capabilities [9,10,11]. The localization of damage using Lamb waves relies on the waveforms generated by their excitation and reception on the surface of plate structures. The propagation theory of elastic waves is always followed throughout the entire transmission and reception process, which is essentially related to mechanics [12,13,14]. The Lamb wave refinement time reversal method was proposed by Kapuria et al. [15] to locate the plate structure without baseline damage and used it to calculate the damage index by calculating the extension packet next to the main wave packet of the reconstructed signal to obtain more accurate damage sensitivity. Marappan et al. [16] employed the gap smoothing method to analyze and identify stratification in conical composite plates. By augmenting the sensitivity of the curvature damage factor (CDF) through the addition of more modes, they effectively reduced experimental uncertainties. However, this method has the problem that the longitudinal sensor grid is relatively rough, which may affect the accuracy of damage detection. A new layered damage detection strategy for structural health monitoring of composite materials based on the inverse finite element method was proposed by Ganjdoust et al. [17]; through the definition of various damage indices, the health of the structure is evaluated in terms of the presence of damage as well as its extent and through-the-thickness position and in-plane size of the damage in laminated composite materials; however, it does not take into account the influence of damage location and different damage shapes on the detection of the algorithm. A damage location method for plate structures based on reflected guided wave coefficient matrix was proposed by Zhang et al. [18], combining the orthogonal matching tracking algorithm (OMP) and elastic wave propagation theory, the reflected wave coefficient matrix (RCM) containing damage information was obtained to predict the specific location of damage reflected signals at different distances.

However, traditional damage location methods encounter challenges related to complex feature signal acquisition and low efficiency in damage localization. To address these issues, the Convolutional Neural Network (CNN) is proposed for damage feature extraction [19], coupled with Long Short-Term Memory (LSTM) networks for predicting damage locations [20]. When applying deep neural networks for research, it is necessary to explore the triangular balance between robustness, accuracy, and fairness. The above three performances were compared against each other and proposed to choose a more cost-effective performance in a specific environment to meet the needs of model application weighed by Li et al. [21]. The Deep Convolutional Neural Network Probabilistic Imaging Algorithm (DCNN-PIA), proposed by Liu et al. [22], leverages the semi-supervised nature of deep learning to overcome issues with feature selection and sensor imbalance. This method offers an automatic and advanced approach for guided imaging, allowing for more intuitive observation of damage locations. However, the deep learning structure monitoring model also needs to manually extract the time coefficient, and there is still a certain gap from the automatic DI extraction. Training Convolutional Neural Networks using efficient hybrid finite element (FE) and ray-based simulations was proposed by Pyle et al. [23] to characterize real defects. The 6-dB descent method is used to determine the size, and a comparison experiment is conducted in four plane wave images of two arrays. The experimental results show that the average error of image size determination based on deep learning is reduced by ±1.05 mm and 9.0° compared with the traditional method. The stratified prediction method of the plate based on low-frequency structural vibration was proposed by Khan et al. [24], the transient response was converted into two-dimensional spectral frame form under the action of short-time Fourier transform (STFT), and discriminant features were extracted from the spectral diagrams and combined with CNN to distinguish various damages. In terms of damage localization, Tabian et al. [25] used passive sensing and CNN for collision detection and localization of complex composites (e.g., composite fuselage panels) and achieved 95% accuracy. However, it only used 96 sets of data for training, which cannot guarantee the usability of the method under large datasets. Mahajan et al. trained the machine learning model by extracting the time domain, frequency domain, and time-frequency domain features of the signal and conducted a total of 672 numerical simulations of different types of damage, severity, and location. The results showed that the method can detect the smallest defect of 5% of the area of the rail head with a thickness of 1 mm [26]. A genetic algorithm to automatically configure the hyperparameters of Convolutional Neural Networks proposed by Johnson et al. [27], by retaining the valid sequence of the parent structure and dynamically adjusting the number of network layers, they achieved recognition accuracies of 99.56%, 84.85%, and 33.3% on the MNIST, CIFAR10, and Caltech256 datasets. Lin et al. [28] proposed a novel method of applying CNN to bridge damage recognition by training on acceleration data from nine measurement points obtained through simply supported beam vibration tests. Their results demonstrated the CNN’s robust performance in damage recognition under noisy and weak excitation environments.

However, employing Convolutional Neural Networks (CNNs) for damage location, as described above, may encounter issues such as gradient explosion [29] during the processing of long time series data. In contrast, Recurrent Neural Networks (RNNs) prove more adept at handling time series data. RNN constitute a category of neural networks designed specifically for processing sequential data. They operate recursively along the evolutionary direction of the sequence, with all recurrent unit nodes interconnected in a chain-like manner [30,31]. Research on RNN commenced in the 1980s and 1990s, evolving into a prominent deep learning algorithm [32,33] in the early 21st century, where the most common recurrent neural networks are Simple Recurrent Neural Network (SRNNs), Gated Recurrent Unit (GRU) recurrent neural network [34], Long Short-Term Memory (LSTM) recurrent neural networks [35], etc. Kuok et al. [36] used Broad Bayesian Learning (BBL) to implement a flat and scalable architecture, improving the fitting ability through incremental expansion without retraining the entire network, reducing computational costs, and the results showed that the predicted modal frequencies were highly consistent with the measured values. The LSTM recurrent neural network effectively addresses the issues of gradient explosion and vanishing gradients encountered when processing long time series data by enhancing the hidden unit structure of conventional recurrent neural networks. Whether it is the complex experimental layout and high accuracy requirements of signal feature extraction that exist in most traditional damage detection methods, or the gradient explosion and gradient vanishing problems that may exist when using Convolutional Neural Networks (CNNs), this paper comprehensively evaluates the advantages and disadvantages of the two, and makes an innovation in the field of damage detection using deep learning methods by integrating the advantages of the CNN and LSTM, enabling the extraction of spatial features of damage signal data from plate structures at different time levels and temporal features from the time dimension of damage signal data. Experimental results demonstrate the effectiveness of this approach on the collected dataset.

The rest of this paper is organized as follows: In Section 2, the CNN-LSTM model is proposed. Section 3 organizes the concrete steps of the experiment and discusses the specific ways of obtaining damage data. The experimental results are compared in Section 4. Section 5 summarizes the full text.

## 2. Based on CNN-LSTM Damage Localization Method

**Figure 1:**
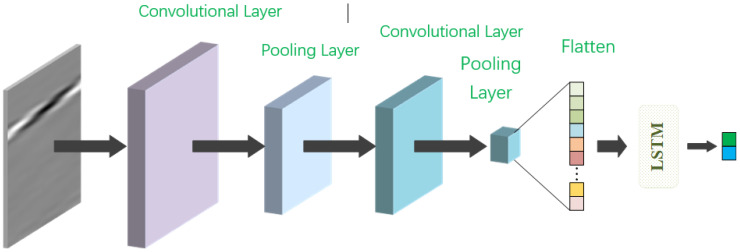
CNN-LSTM model.

The CNN-LSTM model proposed in this paper, as shown in Figure 1, consists of one convolutional layer, one maximum pooling layer, another convolutional layer, another maximum pooling layer, a spreading layer, an LSTM module, and an output layer, in sequence.

The first convolutional layer utilizes 32 convolutional kernels of size 3 × 3 to perform sliding computations on the input data, the 3 × 3 convolution kernel can better extract local spatial features. It calculates the dot product between the input and the convolutional kernels using a default step size of 1 and the Same padding method. Following the first convolutional layer, the pooling layer employs a maximum pooling operation with a pooling region size of 2 and a stride of 2. The activation function between the first convolutional layer and the pooling layer is ReLU.

Subsequently, the second convolutional layer employs 64 convolutional kernels of size 3 × 3, with a default step size of 1 and the Same padding method. The pooling area of the pooling layer following the second convolutional layer has a size of 4 and a stride of 4. ReLU function is again used as the activation function between the second convolutional layer and the pooling layer.

**Figure 2:**
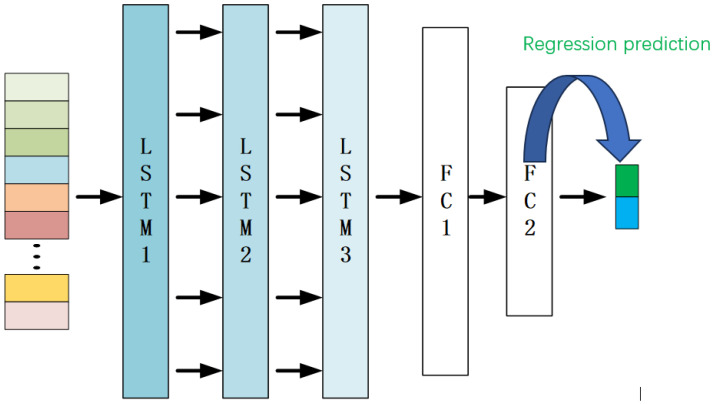
Details of the LSTM module.

The image after the second pooling process is spread and fed into the LSTM module, and the predicted coordinates are finally output. The specific CNN-LSTM model structure proposed in this chapter is illustrated in Figure 1, with Figure 2 providing an expansion of the LSTM module in the model structure shown in Figure 1.

**Figure 3:**
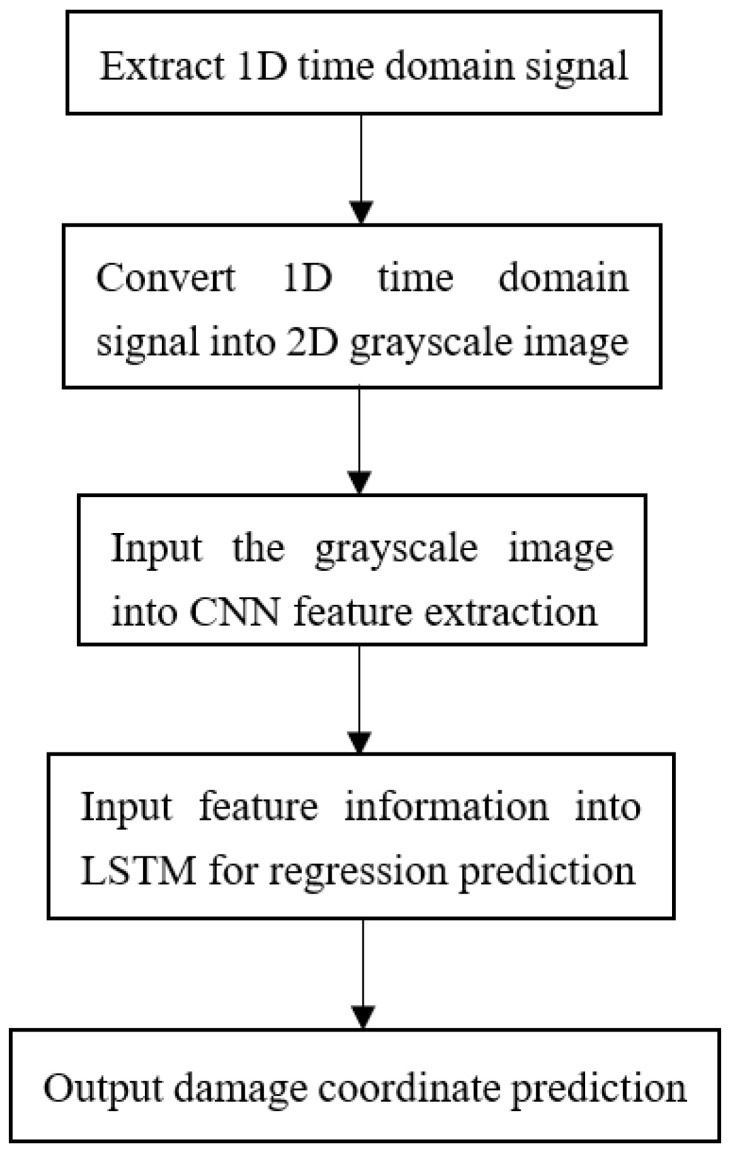
CNN-LSTM damage prediction specific experimental process.

In the designed 2-D CNN-LSTM model, features extracted from the 2-D CNN are utilized as inputs to the LSTM module. This paper employs a three-layer LSTM, followed by two fully connected layers, which are used to connect the final output regression prediction coordinates. It is noteworthy that only the final hidden layer output of the LSTM is fed into the fully connected layer for regression prediction. To mitigate overfitting during training, a dropout method is integrated after each LSTM layer to randomly reduce interlayer neuron connectivity. During experimentation, the CNN-LSTM network undergoes training for 20 epochs. The specific experimental process is shown in Figure 3.

## 3. Experimental Setup

This section describes the experimental preparation, data collection, and analysis of the predicted results.

### 3.1. Data Acquisition

**Figure 4:**
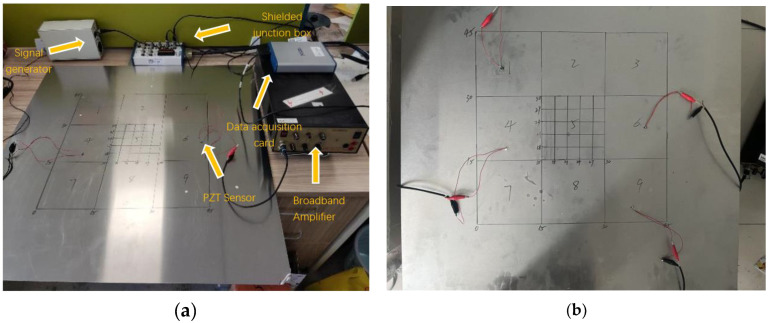
Piezoelectric sensor on aluminum plate damage localization experimental arrangement: (**a**) signal collection experimental equipment, (**b**) PZT sensor layout.

**Figure 5:**
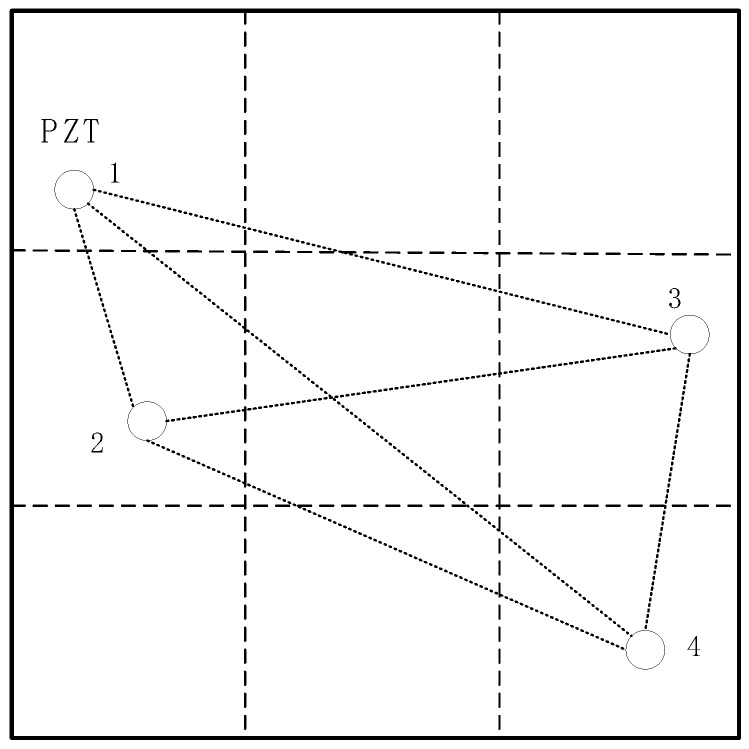
Theoretical schematic diagram of four PZT sensors.

The experimental device in this chapter includes the KH MODEL 7602 broadband produced by Krohn-Hite(USA),amplifier to amplify the output signal of the signal generator, NI USB-4431 data acquisition card produced by National Instruments(USA) to convert the signal received by the sensor into a digital signal, NI BNC-2110 shielded terminal box produced by National Instruments(USA) to reduce the influence of electromagnetic interference on the signal, PSN33 sensor produced by Haiying(China) to stimulate and receive signals, Suin TFG3916A signal generator produced by Suin(China) to generate specific electrical signals, and an aluminum plate with the size of 800.0 mm × 800.0 mm × 2.0 mm. Within the central region of this plate, four piezoelectric lead zirconate titanate (PZT) wafer sensors were strategically positioned, covering an area of 450.0 mm × 450.0 mm. This specific layout was chosen with the aim of mitigating the potential interference of wave propagation caused by an excessive number of sensors within the confined area of the aluminum plate while simultaneously ensuring optimal accuracy. The precise arrangement of these four PZT sensors on the aluminum plate, as well as the subsequent refinement of accuracy, are delineated in Figure 4. Additionally, Figure 5 illustrates a simulation depicting the arrangement of the PZT sensors employed in the experiments detailed within this chapter.

Each of the four PZT sensors functions as an independent signal driver and signal sensor, forming a total of twelve signal transmission–reception channels. Only one PZT sensor operates as a driver at a time, while the other three sensors act as signal receivers, creating twelve distinct signal transmission–reception channels. Specifically, one PZT sensor functions as the driver, and another PZT sensor functions as the sensor, forming a transmit–receive channel.

**Figure 6:**
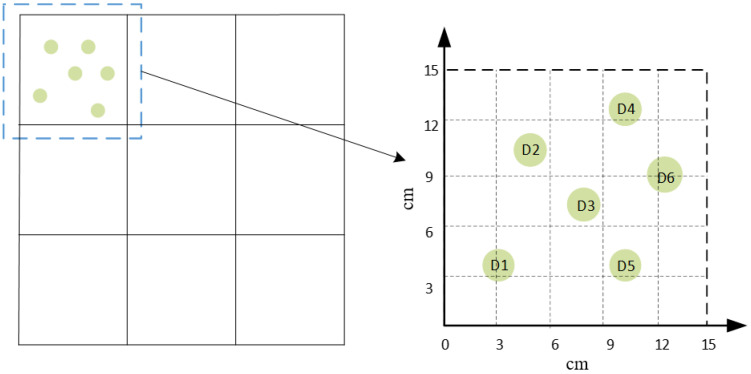
Six damage locations within aluminum compartment 1.

As depicted in Figure 6, since there are many damage locations that need to be simulated during the experiment and considering environmental protection and the fact that signal acquisition on the same aluminum plate can minimize the impact of the aluminum plate itself, the same plate is reused, and the glue pasted on the plate simulates the damage on the aluminum plate. The locations of damages were continuously altered during the experiments to capture data from various positions. For instance, simulations of damage signals were conducted at six different locations within partion 1, denoted as D1, D2, D3, D4, D5, and D6, respectively. The specific coordinates of each damage are presented in Table 1 below.

The data collection method for this experiment involved placing successive 2 cm diameter pieces of mastic on the aluminum plate at different coordinates. Each partition collected 12 × 6 Lamb wave signals, resulting in a total of 54 different damage locations across nine partitions. Additionally, a reference signal was collected in the undamaged state. To mitigate noise effects, the reference signal was collected 10 times repeatedly in the undamaged state, and the average of these 10 signals was taken as the experiment’s reference signal, labeled {0, 0}. The labels for the difference signals in the damaged state corresponded to the coordinates of the damage location. Finally, 660 Lamb wave signals were collected.

To prevent overfitting and enhance the robustness of the final model given the data scale of a single experiment, we opted to conduct five iterations under the same conditions, resulting in the collection of 3300 signals. Subsequently, attempts were made to rotate the aluminum plate by 90, 180, and 270 degrees, with five acquisitions made at each rotation angle to yield 3300 × 4 samples.

Following the four acquisitions in this experiment, a total of 13,200 signal segments were generated, comprising 13,516,800 data points (13,200 × 1024). The dataset was divided into training, validation, and test sets. Specifically, 7920 signal segments were allocated for training, 2640 for validation, and 2640 for testing, as illustrated in Table 2.

### 3.2. Data Processing

Given the excellent performance of Convolutional Neural Networks (CNNs) in image feature extraction, there is a need to convert the 1D signal data collected by sensors into 2D data. Typically, the acquired acoustic signal is processed into a two-dimensional crack image through short-time Fourier transform, while the time-frequency domain map of the signal is artificially generated by selecting the original signal via continuous wavelet transform as the network input. However, these methods encounter issues such as partial information loss and a complex feature extraction process. For instance, they demand a solid theoretical understanding of signal processing, and errors during the two-dimensionalization of the time-domain signal can significantly impact the final experimental outcomes. In this study, we opted for a data pre-processing method that converts the original data into a grayscale map when constructing the input for the CNN model. CNN is naturally adapted to image data. The 2D grayscale image maps the spatial-temporal information of the 1D signal to pixel intensity, preserving the signal amplitude distribution characteristics. Compared with STFT or CWT, which requires manual selection of time-frequency parameters, the grayscale image directly retains the original signal resolution and avoids information loss. This approach effectively preserves the information of the original signal, reduces computational complexity, and transforms the one-dimensional signal into a two-dimensional image matrix suited for CNN processing, with the expectation of achieving higher localization accuracy.

A grayscale map is an image with pixel values represented between 0 and 255, which will reduce the computational effort of subsequent calculations. Any image can then be represented as a grayscale map as long as the pixel values of the individual points can be converted to valid pixel values in the grayscale map. The specific steps of the method for transforming the original Lamb wave signal into a grayscale image are as follows.

**Figure 7:**
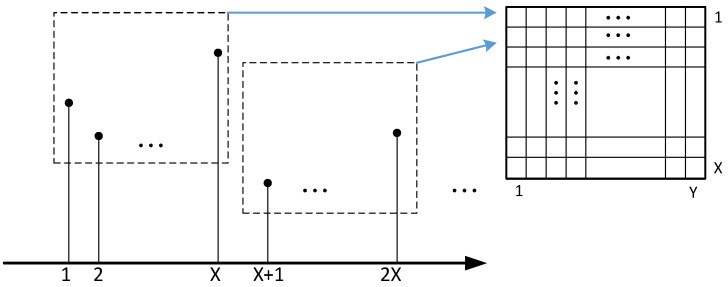
Convert a 1D time-domain signal into a 2D grayscale diagram.

Step 1: As shown in Figure 7, the acquired signal contains X × Y acquisition points. X × Y will also be the size of the final grayscale image, X and Y are the length and width of the grayscale image, respectively, and the size of this value is related to the length of the signal intercepted. For the size of X and Y, it is generally taken that X = Y, which means that the obtained grayscale image is a square image.

Step 2: In order to convert the Lamb wave signal into an image, the amplitude corresponding to each acquisition point of the damage signal is converted to a value somewhere between 0 and 255, specifically the pixel value of the pixel in the grayscale image. The exact conversion is shown in Equation (1) [37].

(1) $$P(i)=round(\frac{255*S\left(i\right)-255*S_{min}}{{S_{max}-S}_{min}})$$

where *S*(*i*) is the amplitude of the *i*-th acquisition point of the Lamb wave signal, *i* = 1, 2, …, X × Y, *S_min_* is the minimum amplitude corresponding to all acquisition points, *S_max_* is the maximum amplitude, *P*(*i*) is the pixel value, and round is a rounding function.

Step 3: Find the pixel coordinates (*m*,*n*), which are calculated as shown in Equations (2) and (3).

(2) m=Floor(i/X)+1

(3) n=Modulo(i/X)

The Floor function is a downward rounding, and Modulo is a modulo operation.

**Figure 8:**
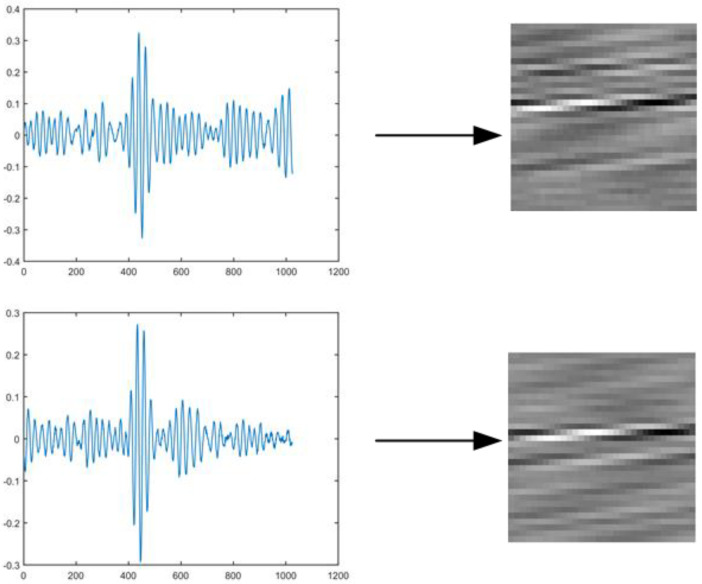
Conversion of 1D time domain signal to 2D grayscale map.

Step 4: Output a grayscale map, where each sample point in the damage signal is converted into the intensity of the corresponding pixel in the grayscale map. In this experiment, a Lamb wave sample is composed of 1024 acquisition points, so the final generated grayscale map is 32 × 32 in size. If different image sizes are used, such as 1 × 1024, the spatial feature extraction capability may be reduced, and only LSTM is used for sequence learning; choosing 1024 × 1 is equivalent to 1D signal input LSTM, which loses the advantage of CNN; choosing 64 × 64 may improve feature resolution but increase computational overhead; choosing 16 × 16 may lead to excessive information compression and reduce damage location accuracy as shown in Figure 8, which gives a specific sample of a 1D difference signal converted into a two-grayscale map.

In this experiment, the signal sampling points are 1024, the grayscale map size is 32 × 32, because the one-dimensional signal is resampled to 32 × 32 = 1024 sampling points, and there is no essential change in time resolution before and after conversion. In special cases, filling a one-dimensional signal of length N into an M×M matrix may require interpolation or downsampling, resulting in sparse pixels or information loss, resulting in reduced resolution. This data preprocessing method effectively reduces the possibility of manual intervention and comprehensively retains the features of the Lamb wave damage signal as input to the neural network for damage location regression diagnosis. This facilitates the implementation of subsequent damage location diagnosis methods and improves the accuracy of damage localization.

### 3.3. Network Parameter Setting

There are many adjustable parameters in neural networks, such as the number of neurons per layer and the learning rate. By adjusting these parameters, the prediction accuracy of the model can be effectively improved. The number and proportion of neuron nodes in the fully connected layers of the LSTM network determines the structure of the network and the prediction results. Therefore, training iterations were performed using the dataset collected in the previous section to determine the optimal number and proportion of nodes in each layer. The hyperparameter settings for the model proposed in this chapter are presented in Table 3.

## 4. Experimental Results

In addition to using the MSE loss function as a model measure in the training of CNN-LSTM models [38], the Mean Euclidean Distance (MED, Mean Euclidean Distance) is also used in this paper to measure the test error, as shown in Equations (4) and (5).

(4) $$MSE=\frac{1}{N}(\sum_{i=1}^{N}\sqrt{(x_{i}-{\hat{x}}_{i}}+(y_{i}-{\hat{y}}_{i}{)}^{2}$$

(5) $$MED=\sqrt{(x_{i}-{\hat{x}}_{i}{)}^{2}+(y_{i}-{\hat{y}}_{i}{)}^{2}}$$

where (${\hat{x}}_{i}$,${\hat{y}}_{i}$)are the predicted coordinates of the damage location, (*x*,*y*) are the true coordinates of the damage, and *N* is the number of samples.

**Figure 9:**
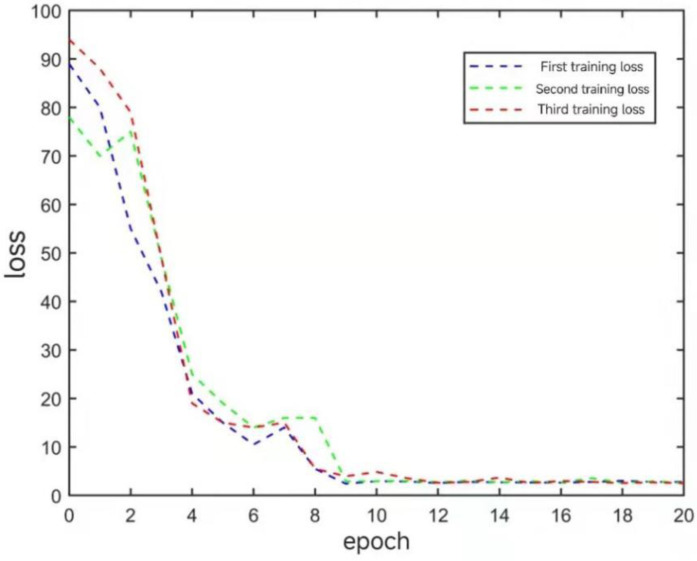
CNN-LSTM model training loss values.

The CNN-LSTM model was trained for 20 epochs per cycle, and the loss variation during training is shown in Figure 9, which shows the change in loss after three repetitions of training. It can be observed that the loss value of the model is relatively high before the training, and the loss value is between 75 and 100 before the training starts. After about 11 epochs of training, the loss tends to stabilize and no longer decreases, and the loss decreases to between 2.4 and 3.0.

In order to study some test cases, the prediction results of the model were displayed. In order to show the uniform selectivity of the experimental aluminum plate machine, a sample was selected in each of the aluminum plates to study the health of the desktop. In addition, the plate was also tested. For better visualization options in the state of visualization, a total of 10 examples are visualized in this section, and the example information is shown in Table 4.

**Figure 10:**
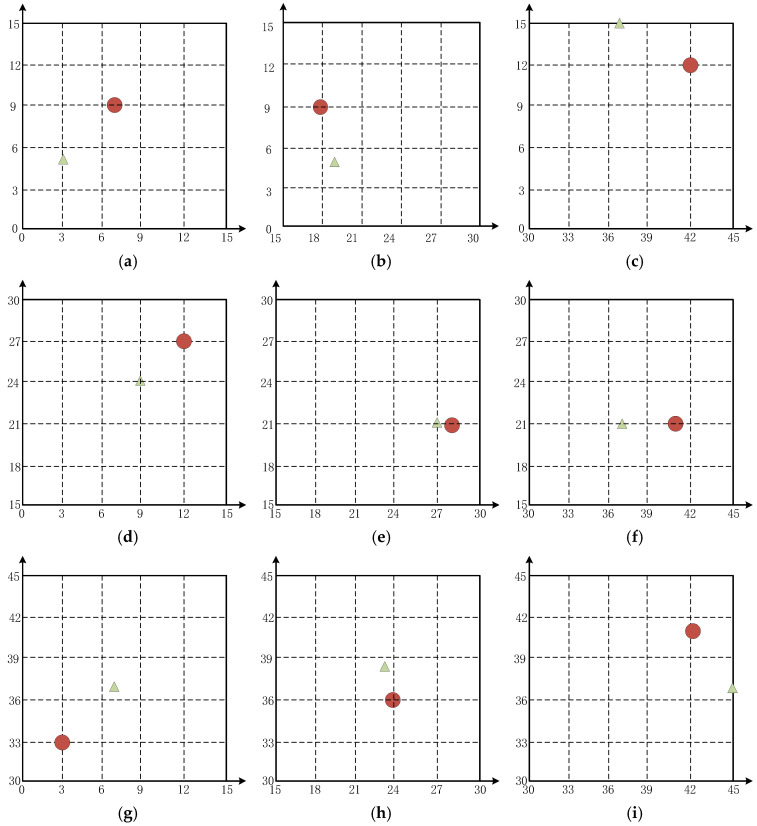
Visualization of randomly selected test samples within nine sub-areas:(**a**) damage is arranged at coordinates (7,9), (**b**) damage is arranged at coordinates (18,9), (**c**) damage is arranged at coordinates (42,12), (**d**) damage is arranged at coordinates (12,27), (**e**) damage is arranged at coordinates (28,21), (**f**) damage is arranged at coordinates (41,21), (**g**) damage is arranged at coordinates (3,33), (**h**) damage is arranged at coordinates (24,36), (**i**) damage is arranged at coordinates (42,41).

As shown in Figure 10, both the predicted and true coordinates of the damage are plotted; in the figure, the red circles represent the true damage coordinates, and the green triangles represent the model’s predicted coordinates.

An alternative measure of CNN-LSTM model performance is as follows, with prediction accuracy defined as the ratio of the number of test samples with errors within a pre-defined threshold to the total number of test samples. When the threshold is 60.0 mm, the accuracy of the test set is 100%; once the threshold reaches 25.0 mm, the accuracy of the test set is 60.5%.

**Figure 11:**
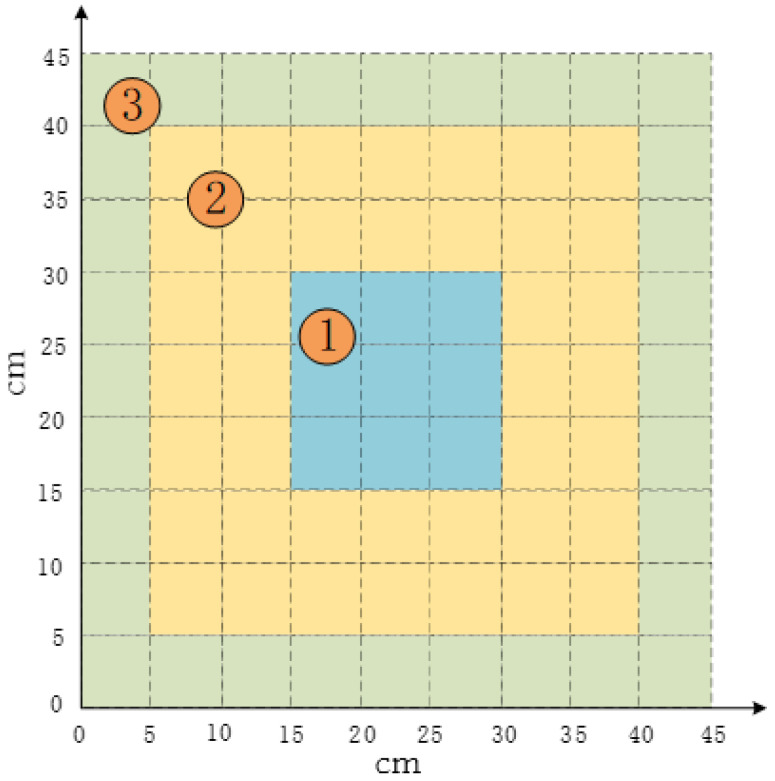
Zoning of the monitoring area.

This section attempts to divide the monitoring area of the aluminum plate into three zones according to the distance from the center of the plate, as shown in Figure 11, which is divided into zone 1, zone 2, and zone 3 in order from near to far. It can be observed from Table 5 that the closer the zone to the center of the aluminum plate, the smaller its average Euclidean distance, indicating the overall prediction of the model for the regression prediction of the coordinates of the damage location on the aluminum plate. Where the damage location is in the center of the monitoring area, it is more effective than the accuracy of the model for the prediction of the damage location coordinates on the aluminum plate.

## 5. Discussion

### 5.1. Algorithm Performance Comparison

To further validate the performance of the proposed method, this section compares two other methods: MLP [24,25] and DI combined with 1-D CNN [26]. In the MLP method, a multilayer perceptron containing two fully connected hidden layers with 512 and 256 nodes, respectively, was constructed and trained on the input difference signal. As can be observed from Table 6, the MLP performed much worse than the CNN-LSTM model, with the worst localization accuracy of the three methods compared; this is because the limited training data in this experiment was not sufficient to construct a good MLP model with good results and poor predictive regression accuracy, with a MED of 12.70 cm. Damage indices are commonly used features in damage signal processing, and several forms of damage indices have been proposed to reflect the effect of damage on Lamb wave signals, such as spatial phase difference, intercorrelation damage factor, etc. This paper also investigates the performance of the method using DI combined with 1-D CNN. The reason for using 1-D CNN is that this method usually involves connecting the calculated multiple damage index features into a one-dimensional vector as input, and the regression accuracy of the model in this method is directly related to the DI used, requiring some prior knowledges to extract the damage index from the complex wave signal. The results of the comparison of the three methods are presented in Table 6.

### 5.2. Impact of Different Damage Counts, Sensor Numbers, and Location

Considering that the arrangement of the sensors and the number of sensors may also cause a large prediction error in the model, four sensor layouts with different numbers and different arrangements were proposed to verify their influence on damage location.

**Figure 12:**
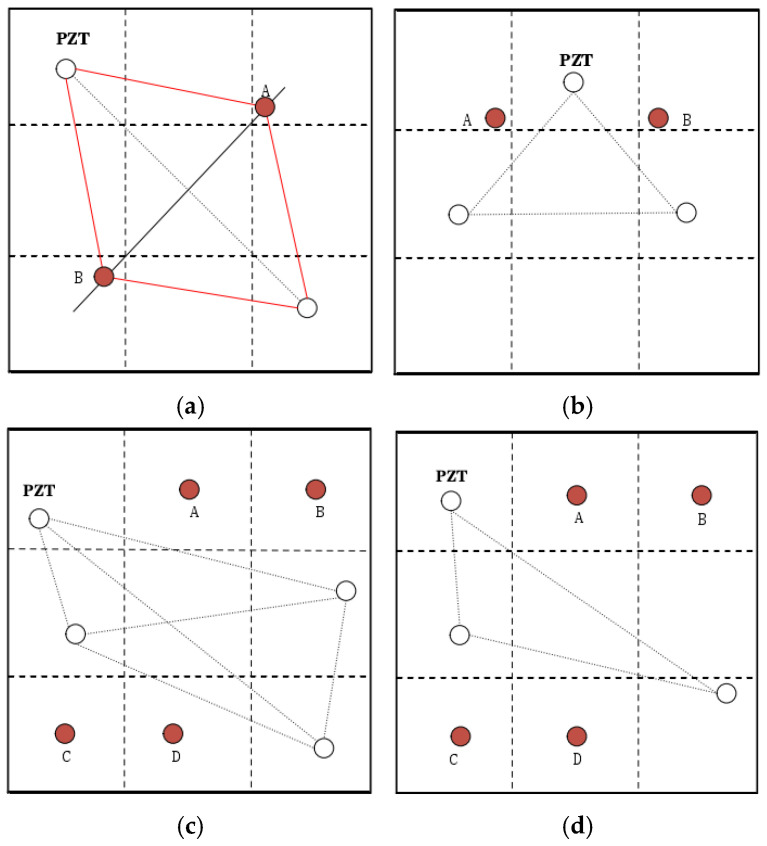
Different sensor numbers and locations: (**a**) symmetrical damage and sensor placement, (**b**) the damage is arranged symmetrically about the midline of the sensor arrangement, (**c**) asymmetric damage and sensor placement, (**d**) asymmetric sensor arrangement.

As shown in Figure 12, in Figure 12a, two sensors are arranged in the 1st and 9th partitions, and although damage A and damage B are distributed in different partitions, and damage C and damage D are placed in the 7th and 8th areas respectively.the connection between the two sensors is symmetrical, which makes them easy to confuse in feature extraction, and the damage localization rate is very low. In Figure 12b, the three sensors are arranged on the aluminum plate in an isosceles triangle pattern, and damage A and damage B are symmetrical in the midline of the isosceles triangle, and the signals collected at the two damage sites easily cause the same waveform, which affects the accuracy of damage location. In Figure 12c, the four sensors are asymmetrical with each other, which is significantly improved compared with the arrangement of Figure 12a,b. Compared with Figure 12b, the Figure 12d symmetrical arrangement is broken, and there are still significant differences in the waveform between the four damages, and the accuracy of the damage is also significantly improved.

**Figure 13:**
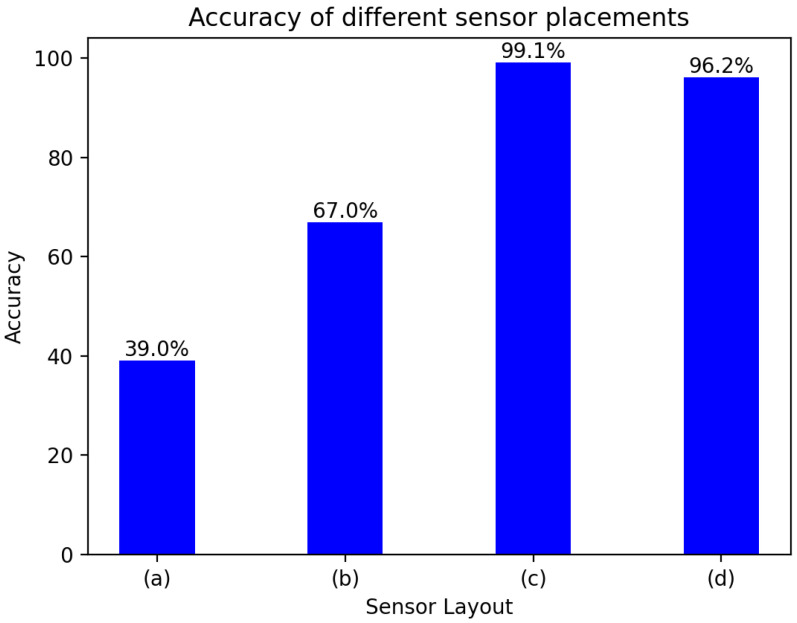
Comparison of accuracy of the four permutations:(**a**) symmetrical damage and sensor placement, (**b**) the damage is arranged symmetrically about the midline of the sensor arrangement, (**c**) asymmetric damage and sensor placement, (**d**) asymmetric sensor arrangement.

According to Figure 13, the location and number of sensors have a great impact on the accuracy of damage location. The accuracy of damage location is very low due to the symmetrical arrangement of the two arrangement methods of (a) and (b), and by changing the symmetry of the arrangement, the accuracy of damage localization was increased from 67.00% to 96.20% of (d), and the accuracy of the accuracy is further increased to 99.10% by increasing the number of sensors in the (d) arrangement mode.

## 6. Conclusions

In this paper, a CNN-LSTM based neural network model is proposed for damage localization of plate structures. The joint CNN and LSTM fully combines the advantages of CNN and LSTM, which can extract both the spatial features of the damage feature signal data of plate structures at different time levels and the temporal features of the damage feature signal data in the time dimension. Recognizing that CNN is one of the best feature extraction methods in the field of image processing and can greatly improve the accuracy of image data classification, we chose to use the original one-dimensional signal data collected in this experiment. We obtained the difference signal by calculating it against the averaged baseline signal and then converted this difference signal into a two-dimensional image, so that CNN can better extract the high-dimensional features of the data from the signal. The CNN extracted features are then input into the LSTM, and the damage prediction is carried out through the unique forgetting mechanism and memory mechanism in the LSTM, and the regression localization experiments are carried out on the damage location on the aluminum plate. In the future, in order to further improve the accuracy of damage diagnosis, the attention mechanism will be considered to improve the feature extraction ability of damage information, and the generalization ability of the model will be improved by increasing the size and diversity of the dataset.

## Figures and Tables

**Table 1:** Coordinates of the six selected damage locations within aluminum compartment 1.

Damage	Coordinates	Damage	Coordinates
D1	(3,4)	D4	(10,13)
D2	(5,11)	D5	(10,4)
D3	(8,8)	D6	(13,9)

**Table 2:** Settings for experimental data.

Total Data	Training Set	Validation Set	Test Set
13,200	7920	2640	2640

**Table 3:** CNN-LSTM hyperparameter settings.

Category	Parameter Selection
Convolution kernel for convolution layer 1	Size: 3 × 3 Number of pieces: 32
1st pooling layer	Size: 2 Step length: 2
Convolution kernel for convolution layer 2	Size: 3 × 3 Number of pieces: 64
2nd pooling layer	Size: 4 Step length: 4
Activation functions	ReLU
LSTM layer	3
1st fully connected layer	512
2nd fully connected layer	256
Learning Rate	0.0009

**Table 4:** Specific information on the visualization sample.

No.	Real Coordinates	Forecast Coordinates	Euclidean Distance (ED) (cm)
a	(7,9)	(3,5)	5.67
b	(18,9)	(19,5)	4.12
c	(42,12)	(37,15)	5.83
d	(12,27)	(9,24)	4.24
e	(28,21)	(27,21)	1.00
f	(41,21)	(37,21)	4.00
g	(3,33)	(7,37)	5.67
h	(24,36)	(23,38)	2.24
i	(42,41)	(45,37)	5.00
j	(0,0)	(0,0)	0.00

**Table 5:** Average Euclidean distances for the three partitions in the test set.

Real Damage Areas	Mean Euclidean Distance (MED) (cm)
1	1.14
2	2.34
3	5.42

**Table 6:** Comparison of the effects of the three damage detection methods.

Methods	Mean Euclidean Distance (MED) (cm)
CNN-LSTM	3.42
MLP	12.70
DI and 1-D CNN	6.75

## Data Availability

The raw data supporting the conclusions of this article will be made available by the authors on request.
